# Multi-Omics Analyses Unravel Metabolic and Transcriptional Differences in Tender Shoots from Two *Sechium edule* Varieties

**DOI:** 10.3390/cimb45110568

**Published:** 2023-11-13

**Authors:** Zhihui Du, Fei Qu, Chaojun Zhang, Zhilin Chen, Yurong Li, Linhong Wen

**Affiliations:** Guizhou Horticulture Institute, Guizhou Academy of Agricultural Sciences, Guiyang 550009, China; dzh8928088@163.com (Z.D.); 18798006209@163.com (F.Q.); fqzcj@126.com (C.Z.); chenzhilin@126.com (Z.C.); hong1521@163.com (L.W.)

**Keywords:** *Sechium edule*, chaylte vines, flavonoids, metabolomics, transcriptomics

## Abstract

Chaylte vine, the tender shoot of *Sechium edule*, is popular among vegetable consumers because of its high nutritional content, crisp texture, and unique flavor. Existing studies on the nutrient composition of chaylte vines are mostly simple chemical determinations, which have limited the breeding of specialized cultivars and the development of related industries. Using metabolomics combined with transcriptomics, this study analyzed the metabolic characteristics and related molecular mechanisms of two common varieties of chaylte vines: green-skinned (SG) and white-skinned (SW). Between the two varieties, a total of 277 differentially accumulated metabolites (DAMs) and 739 differentially expressed genes (DEGs) were identified. Furthermore, chemical assays demonstrated that the SW exhibited a higher total flavonoid content and antioxidant capacity. In conclusion, it was found that the SG samples exhibited a higher diversity of flavonoid subclasses compared to the SW samples, despite having a lower total flavonoid content. This inconsistent finding was likely due to the differential expression of the phenylalanine ammonia-lyase (PAL) and chalcone synthase (CHS) genes in the two varieties. These results laid the foundation for investigating the mechanisms involved in flavonoid regulation and the breeding of specialized *S. edule* cultivars for chaylte vine production.

## 1. Introduction

*Sechium edule* (Jacq.) Swartz, a perennial persistent climbing herb of the genus *Sechium* in the family Cucurbitaceae, was originally from Mexico and other Central and South American countries [1]. It was introduced to China in the early 19th century and is now widely cultivated in Guangdong, Yunnan, Zhejiang, Fujian, and Taiwan [2]. Its edible fruit, popularly known as chayote, has the advantages of fast growth and high yield, making it one of the important vegetables in the South China region [3]. In addition to the fruit, the tender shoot, known as the chaylte vine, is also edible [4]. The chaylte vine is rich in vitamins, carotenoids, flavonoids, and mineral elements. The vine’s crisp texture and unique flavor make it suitable for stir-fried or blanched food, therefore gaining popularity in Southwest China and Southeast Asian countries [4,5].

In recent years, researchers have conducted a series of studies on the nutritional composition of chaylte vines [6]. The studies demonstrated that chaylte vines contain a significant amount of protein, fat, fiber, vitamins (A, B1, B2, B3, C, and E), carotenoids, flavonoids, and mineral elements (calcium, iron, and phosphorus) [3,7]. The protein content of the chaylte vine was found to be 2.3 times higher than that of the chayote (fruit). Moreover, the chaylte vine exhibited higher levels of total flavonoids, total phenols, proanthocyanidins, β-carotene, vitamin E content, and antioxidant capacity compared to the chayote [8]. Additionally, the chaylte vine demonstrated potent α-amylase inhibitory activity, suggesting its potential for lowering blood glucose levels [4]. These findings provided a general understanding of the nutritional content of chaylte vines. However, in contrast to the advancements made in studying the nutrient composition, molecular mechanisms, pharmacological effects, and development of chayotes, the studies on chaylte vines have focused solely on compositional determination [9,10,11]. This limitation has hindered the genetic selection and exploitation of chaylte vines. For example, to the best of our knowledge, there are no specialized cultivars of chaylte vines available on the market, and most existing chaylte vines were produced by directly altering the cultivation of fruiting *S. edule*.

In order to systematically unravel the metabolite profile of the chaylte vine, as well as provide a reference for its varietal selection, two common varieties of chaylte vines were selected as test materials in this study. While the two selected varieties of *S. edule* exhibited differences in the fruit rind color, discerning phenotypic differences in the chaylte vines was not possible by the naked eye. To analyze the differences in the metabolites and related metabolic pathways between the two chaylte vine varieties, widely targeted metabolomics technology was employed. Simultaneously, RNA-seq sequencing was conducted, and the sequencing data were jointly analyzed with the metabolomics data to identify the genes associated with the differential accumulation of metabolites. This analysis provides theoretical references for investigating the production of differentially accumulated metabolites between different varieties and the breeding of chaylte vines.

## 2. Materials and Methods

### 2.1. Plant Sample Preparation

**Figure 1:**
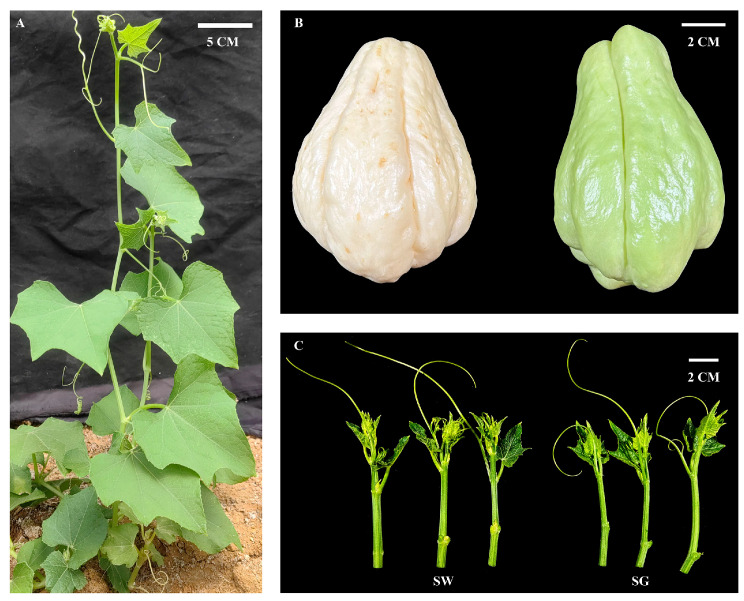
Morphological characteristics of chaylte vine and *S. edule*. (**A**) Growing state of *S. edule* at sampling. (**B**,**C**) Two varieties of chaylte vines: green-skinned (SG) and white-skinned (SW), and their corresponding fruits.

In the spring of 2023, green-skinned (SG) and white-skinned (SW) varieties of *S. edule* were cultivated in the germplasm garden (Guiyang, China). When the main stem of the plants reached a height of 60 cm, the shoot tips located approximately 10 cm from the stem tip were collected. These shoot tips were then referred to as chaylte vines (Figure 1). A total of nine replicates were taken for each variety of chaylte vine. These replicates were divided into three categories: metabolomics analysis, transcriptomics analysis, and chemical experimentation. Specifically, three replicates were allocated for each category. All the samples were promptly frozen in liquid nitrogen and subsequently stored in an ultra-low temperature freezer (−80 °C) until further use.

### 2.2. Widely Targeted Metabolomics Analysis of Metabolites between Two Varieties of Chaylte Vines

The preliminary preparation of metabolomics was carried out in accordance with established protocols at Metware Biotechnology Co., Ltd. (Wuhan, China). The strategy of the widely targeted metabolomics based on ultra-high performance liquid chromatography-tandem mass spectrometry (UPLC-MS/MS) assay referred to the published research by Huazhong Agricultural University [12].

The mass spectrometry data were processed using the software Analyst v1.6.3. Metabolite identification was accomplished by comparing the mass spectra with the MWDB database. MRM was used to perform qualitative analysis of the raw data. To normalize the quantitative data, an internal standard (methanol) was used. Afterwards, the metabolomics data were analyzed using the OPLS-DA model [13]. From the OPLS-DA results, VIP (variable importance for projection) values of the metabolites were extracted. The differentially accumulated metabolites (DAMs) between the groups were determined using VIP (VIP ≥ 1) and Log2 fold change (|Log2 fold change | ≥ 1.0).

### 2.3. RNA-Seq and Transcriptome Data Analysis

The samples were first subjected to total RNA extraction using Trizol (Invitrogen, Waltham, MA, USA). The quality control of the extracted RNA was evaluated using a Qubit^®^2.0 Fluorometer (Life Technologies, Carlsbad, CA, USA) and further assessed through 1% agarose gel electrophoresis. Subsequently, a NEBNext^®^ UltraTM RNA Library Prep Kit for Illumina^®^ (NEB, Ipswich, MA, USA) was employed to construct sequencing libraries while following the manufacturer’s recommendations. The AMPure XP system (BeckmanCoulter, Boston, MA, USA) was then utilized to screen cDNA fragments ranging from 250 to 300 bp, followed by polymerase chain reaction (PCR) amplification. Ultimately, RNA-Seq was executed on the Illumina Novaseq platform (Illimina, San Diego, CA, USA).

The raw Illumina reads underwent preprocessing with fastp, which involved trimming adapters [14]. The low-quality sequences were removed based on two criteria: N base content exceeding 10% or base content (Q ≤ 20) exceeding 50%. Subsequently, hisat2 was employed to build the index of the reference genome of *S. edule*, and all the filtered clean reads were aligned to this index [9,15]. In parallel, novel transcripts were predicted using stringtie, while itak was used for the prediction of transcription factors [16,17]. To functionally annotate the genes, they were compared against several frequently used databases, including NR (NCBI nonredundant), KEGG (Kyoto Encyclopedia of Genes and Genomes), and so on.

The gene expression was calculated using the fragments per kb of transcript per million (FPKM) method [18]. To judge the significance of the differentially expressed genes (DEGs) between the varieties of chaylte vines, we corrected the *p*-value to establish a false discovery rate (FDR) threshold of FDR < 0.05. Additionally, we required an absolute value of log2 (fold change) > 1; DESeq2 was employed for this analysis [19]. Furthermore, clusterProfiler was used to analyze the statistical enrichment of the differentially expressed genes (DEGs) in the KEGG pathways [20].

### 2.4. Combined Analysis of Metabonomics and Transcriptomics

Pearson correlation analysis was conducted to determine the quantitative values of all the genes and metabolites in the samples. The resulting correlations were then presented in a nine-quadrant diagram. The relationships with a coefficient absolute value greater than 0.975 were considered crucial and were shown in the network.

### 2.5. Quantitative Real-Time PCR (qRT-PCR)

First, the PrimescriptRT reagent kit with a gDNA eraser (TaKaRa, Tokyo, Japan) was used to perform the first-strand cDNA synthesis, following the manufacturer’s instructions. QRT-PCR was then conducted on an ABI 7500 system (Applied Biosystems, Foster City, CA, USA) using TB Green^®^ Premix Ex Taq™. The 18s rRNA gene was utilized as an internal control, and three biological replicates were included in each analysis. The relative expression levels were determined using the 2^−ΔΔCt^ method [21]. The primer sequences can be found in the Supplementary Table S1.

### 2.6. Determination of Total Flavonoid Content and Protective Enzyme Activity

The determination of the total flavonoid content in the chaylte vines was conducted using the aluminum nitrate colorimetric method. A standard curve was drawn using 0.528 mg/mL of rutin. A fresh sample weighing 0.5 g was taken from each sample and extracted using a 60% ethanol solution through reflux. The solution was then filtered, evaporated to dryness, and fixed to a volume of 25 mL. Subsequently, 4 mL of each sample solution was mixed with 0.5 mL of 5% NaNO2 solution, and the absorbance at 510 nm was measured. From the standard curve, the content of rutin in the sample solution was determined, and, subsequently, the content of total flavonoids in the sample was calculated in mg/g (fresh weight).

The activity of superoxide dismutase (SOD) was determined using the nitro blue tetrazolium (NBT) reduction method. The assay tubes and control tubes were measured for light absorption at 560 nm, and one unit of SOD activity was defined as the amount of enzyme required to achieve 50% inhibition of the NBT reduction reaction. The guaiacol method was employed to assess peroxidase (POD) activity. One unit of POD activity was defined as a 0.01 change in absorbance at 470 nm per minute per g of tissue per mL of the reaction system. The malondialdehyde (MDA) content was estimated using the thiobarbituric acid test (TBA). The reaction product was utilized to measure absorbance values at 450, 532, and 600 nm, which were then employed to calculate the MDA content in nmol/g. The detailed methods, referenced in published reports, can be found in Supplementary Table S2 [22].

A *t*-test analysis was conducted to calculate the significant differences between the two sample groups. GraphPad Prism 9 was utilized to calculate and illustrate the results of the statistical tests. One star indicated a *p*-value below 0.05, while two stars indicated a *p*-value below 0.01.

## 3. Results

### 3.1. Metabolic Analysis between Two Varieties of Chaylte Vines

**Figure 2:**
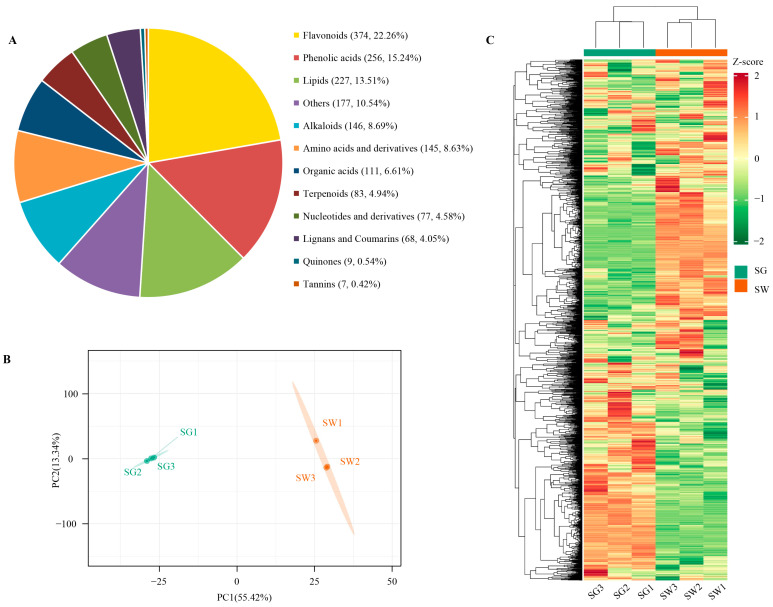
(**A**) Classification and proportion of 1680 metabolites detected in chaylte vines. (**B**,**C**) PCA and hierarchical clustering heatmap of metabolites of two varieties of chaylte vines, respectively.

The analysis of the metabolomics revealed that a total of 1680 components were detected from two different varieties of chaylte vines using UPLC-MS/MS (Figure 2A; Figure S1; Figure S2). These components can be classified into 12 distinct classes, which include flavonoids, phenolic acids, lipids, alkaloids, amino acids and derivatives, organic acids, terpenoids, nucleotides and derivatives, lignans and coumarins, quinones, tannins, and others. Principal component analysis (PCA) of the 1680 metabolites demonstrated that the two chaylte vine varieties could be entirely distinguished. Additionally, the hierarchical clustering heatmap corroborated these findings, further indicating the presence of differences between the metabolites of the two sample groups (Figure 2B,C).

The classes with the highest number of components detected were flavonoids (374, 22.26%), phenolic acids (256, 15.24%), and lipids (227, 13.51%). These three metabolite classes combined accounted for more than half of all the detections and can be considered the most important components of chaylte vines. Specifically, the green-skinned variety (SG) contained eight unique metabolites consisting of five flavonoids (quercetin-5,4′-di-O-glucoside, vitexin-7-O-(6″-feruloyl)glucoside, kaempferol-3-O-arabinoside, Okanin-4′-O-glucosyl-O-glucoside, and okanin-3′,4′-di-O-glucoside*), two phenolic acids (calceolarioside A and 2-hydroxy-3-phenylpropanoic acid), and one organic acid (nonanoic acid), while the white-skinned variety (SW) had four unique metabolites, including two flavonoids (choerospondin and Limocitrin-3-O-galactoside), one phenolic acid (isoeugenol), and one alkaloid (4-aminoindole) (Table S3). In contrast, the number of detected metabolites shared by the two groups of samples, SG and SW, was as high as 1668. This indicates that there is no significant difference between the two varieties in terms of metabolite species. Therefore, it can be hypothesized that the differences in the metabolite levels between the two sample groups primarily reflect variations in metabolite accumulation rather than species composition.

### 3.2. Differentially Accumulated Metabolites between Two Varieties of Chaylte Vines

**Figure 3:**
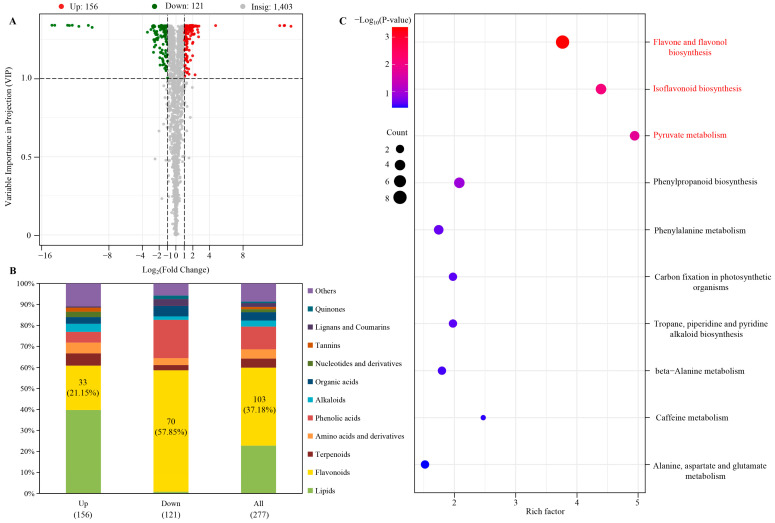
(**A**) Volcano plot of DAMs between two varieties of chaylte vines. (**B**) Classification and proportion of DAMs. (**C**) KEGG enrichment analysis of DAMs. Pathways with significant enrichment are highlighted in red.

OPLS-DA was performed to screen the DAMs between the two groups of samples (SG vs. SW) in order to further reveal the differences in the accumulation of metabolites between the two varieties of chaylte vines. The R2X value for the OPLS-DA model was greater than 0.6, and the R2Y and Q2 values were greater than 0.95 (Figure S3). These results suggested that the model was not overfitted and possessed good discriminatory ability for the samples in this study. A total of 277 DAMs were identified between the SG vs. the SW, with 156 up-regulated and 121 down-regulated DAMs in the SW compared to the SG (Figure 3A; Table S4).

The most abundant up-regulated differentially accumulated metabolites (DAMs) in the SW samples were lipids (62, 39.7%), followed by flavonoids (33, 21.2%). On the other hand, the down-regulated DAMs were mostly composed of flavonoids (70, 57.85%), accounting for approximately 60% of the total down-regulated DAMs, followed by phenolic acids (22, 18.2%). Additionally, flavonoids constituted the largest number of total DAMs (103, 37.2%) (Figure 3B; Table S4). These findings signify that the differential accumulation of flavonoid metabolites played a crucial role in the metabolic variances between the two chaylte vine varieties, SW and SG.

The KEGG pathway annotation and enrichment analysis of 277 DAMs were performed to gain insight into the metabolic pathways involved. The results, shown in Figure 3C, revealed three significantly enriched pathways, which were screened based on a *p*-value of <0.05. These pathways were identified as the flavone and flavonol biosynthesis pathway (ko00944, rich factor: 3.77), isoflavonoid biosynthesis pathway (ko00943, rich factor: 4.40), and pyruvate biosynthesis pathway (ko00620, rich factor: 4.94) (Figure 3C). Further analysis indicated that the biosynthesis and differential accumulation of flavonoids had a significant impact on causing metabolic differences between the SG and SW samples.

### 3.3. Analysis of Transcriptomics between Two Varieties of Chaylte Vines

Two groups of samples, SG and SW, were collected to explore the genes and related pathways involved in the metabolic differences between two varieties of chaylte vines. RNA-seq using the paired-end sequencing technique was performed on these samples. In total, 369,729,870 sequencing data of raw reads were obtained from six cDNA libraries. After quality control and data filtering, 191,062,462 and 167,393,980 high-quality clean reads were acquired from the SG and SW samples, respectively. The Q30 percentage of clean bases for both samples was 94.40% and 94.49%, respectively (Table 1). Out of the clean reads, 341,964,808 (95.40%) were mapped uniquely against the reference genome. Furthermore, all of the clean reads were used for de novo assembly, resulting in the generation of 3096 novel transcripts using Trinity software (v2.15.1).

**Figure 4:**
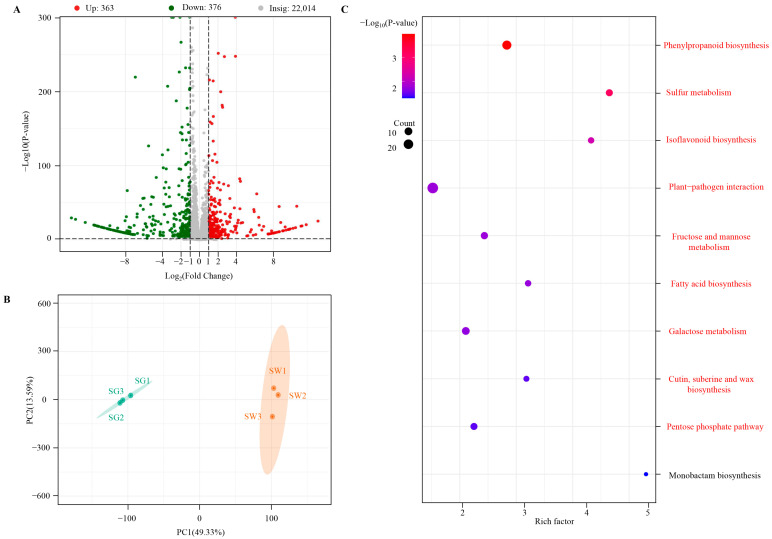
(**A**) Volcano plot of DEGs between two varieties of chaylte vines. (**B**) PCA of genes of two varieties of chaylte vines. (**C**) KEGG enrichment analysis of DEGs. Pathways with significant enrichment are highlighted in red.

The FPKM method was used to determine the expression level of the genes. PCA analysis of the gene expression levels revealed a significant separation between the SW and SG samples with some aggregation observed within each sample (Figure 4B). This suggests that the sequencing results are suitable for the subsequent analysis of differential gene expression. By comparing the two samples, a total of 739 DEGs were identified. Specifically, 363 genes were up-regulated and 376 genes were down-regulated in the SW group compared to the SG group (Figure 4A; Table S5).

KEGG pathway enrichment analysis was performed to determine the functions associated with the changes in DEGs between the two groups of samples. The analysis revealed that a total of 140 KEGG pathways showed hits with DEGs, out of which nine pathways were significantly enriched (*p*-value < 0.05), as shown in Figure 4C. Two of these enriched pathways were specifically related to flavonoid biosynthesis, namely phenylpropanoid biosynthesis (ko00940) and isoflavonoid biosynthesis (ko00943), with rich factors of 2.83 and 4.33, respectively. Among these two pathways, ko00940, which serves as the initiation of flavonoid biosynthesis, was found to be the most significantly enriched pathway. These findings suggest that the genes involved in ko00940 and other flavonoid-related pathways may play a significant role in the metabolic differences observed between the two varieties of chaylte vines, particularly in the context of flavonoid production.

### 3.4. Differentially Expressed Genes in Flavonoid-Related Pathway

**Figure 5:**
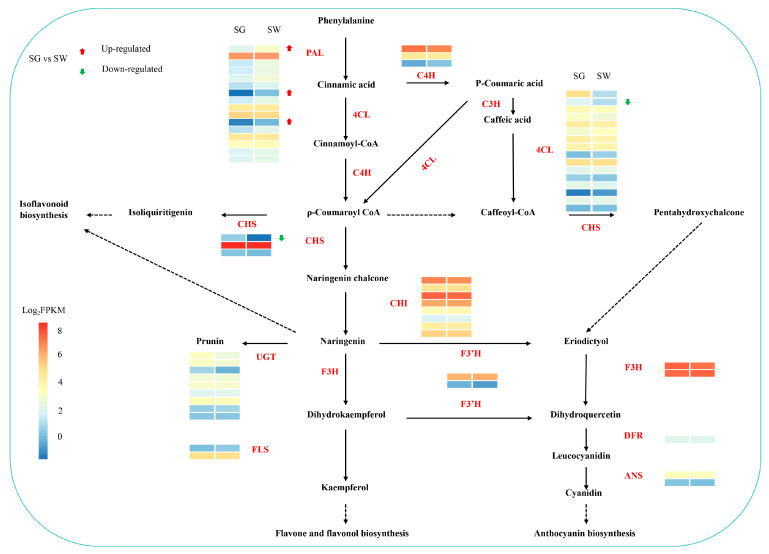
The expression profiles of genes encoding enzymes involved in flavonoids between two varieties of chaylte vines. Dotted lines represented multiple steps. Abbreviations are shown in Table S6.

The metabolomics and transcriptomics analyses indicated variations in the flavonoid metabolite content and the expression levels of genes related to the flavonoid biological pathway between two varieties of chaylte vines. Consequently, we conducted a gene expression analysis to further investigate the patterns of flavonoid biosynthesis (Figure 5).

In the chaylte vines, the expression of downstream genes involved in flavonoid biosynthesis was higher, such as the genes encoding CHI and F3H in both the SG and SW samples, compared to the genes of the upstream pathway. The preliminary finding suggests that the genes encoding these enzymes play a significant role in the flavonoid biosynthesis of chaylte vines. However, there was no significant difference in the expression of these genes between the two groups of samples, indicating that they may not be the key genes leading to the differential accumulation of flavonoids in the SW and SG samples. Notably, the expression of upstream regulatory genes differed more significantly between the two groups of samples. Specifically, three genes responsible for encoding PAL were significantly up-regulated, while one gene each from the 4CL and CHS gene families was significantly down-regulated in the SW samples. It is possible that the differential expression of these upstream structural genes is responsible for the variations in the flavonoid metabolite production between the two varieties of chaylte vines.

**Figure 6:**
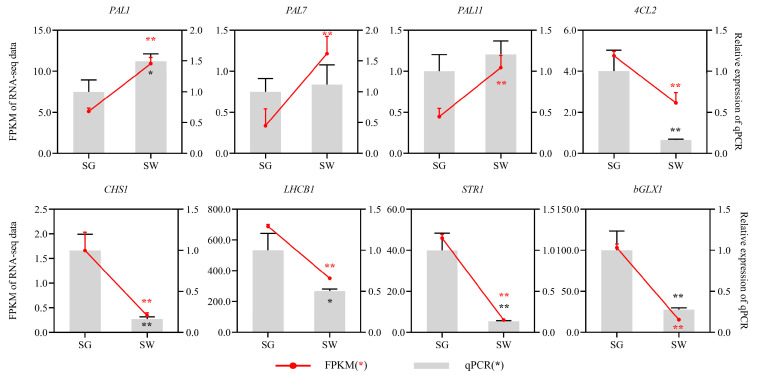
Expression patterns of genes as verified by qRT-PCR. * *p*-value was less than 0.05; ** *p*-value was less than 0.01. LHCB, light-harvesting chlorophyll-binding protein; STR, strictosidine synthase; bGLX, beta-glucosidase. Other abbreviations are shown in Table S6.

Overall, qRT-PCR analysis was conducted to verify the accuracy of the RNA-Seq data using the five key DEGs mentioned in addition to three other genes. The qRT-PCR results indicated that these genes exhibited the same expression trends as the RNA-seq data, providing evidence of the high level of reliability of the RNA-seq data (Figure 6).

### 3.5. Combined Analysis of Metabonomics and Transcriptomics

**Figure 7:**
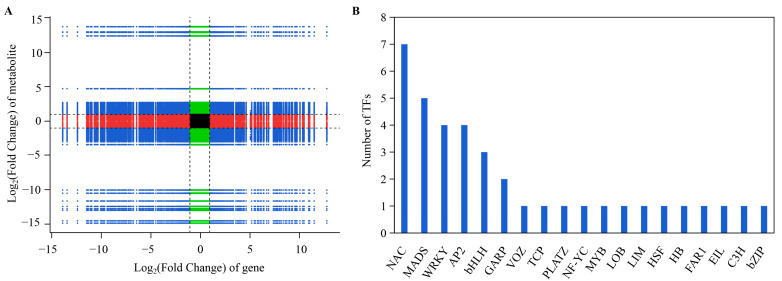
(**A**) Nine-quadrant diagram of correlation between genes and metabolites. (**B**) Transcription factors (TFs) in quadrants 1, 3, 7, and 9.

The quantitative values of all the genes and metabolites in all the samples were analyzed for correlation in order to explore the potential regulatory relationship between the flavonoids and related genes (Figure 7A). The diagram is divided into quadrants 1–9 from left to right, top to bottom, using the black dashed line as the boundary. Within each quadrant, the Pearson correlation coefficients between each gene and metabolite were greater than 0.80, and the *p*-value was less than 0.05. Consistency in the differential expression patterns of the genes and metabolites was observed in quadrants 3 and 7, suggesting a positive regulation of genes that might be responsible for the cumulative changes in the metabolites. Conversely, the expression patterns of the genes and metabolites in quadrants 1 and 9 showed a negative correlation, implying that the cumulative changes in the metabolites might be caused by negative regulation of the genes. Weak correlations between the genes and metabolites were observed in the remaining quadrants.

The data were exported for quadrants 1, 3, 7, and 9 where the genes were strongly correlated with the metabolites, which contained 295 metabolites (including 108 flavonoids) and 753 genes. In this dataset, all five DEGs (three PALs, one 4CL, and one CHS) in the flavonoid biosynthesis pathway were included. Additionally, 38 transcription factors, including NAC, MADS, WRKY, and MYB, were found in this dataset (Figure 7B; Table S7).

### 3.6. Construction of Transcriptional Metabolic Regulatory Network of Flavonoids in Chaylte Vines

A correlation analysis of the flavonoid DAMs and DEGs in quadrants 1, 3, 7, and 9 was carried out to further reveal the correlation between the flavonoids and genes in the chaylte vines. After a rigorous selection process, a correlation network was established to find the regulatory correlation between the metabolites and genes involved in flavonoid biosynthesis. This analysis also aimed to explore candidate TFs for regulating flavonoid biosynthesis.

**Figure 8:**
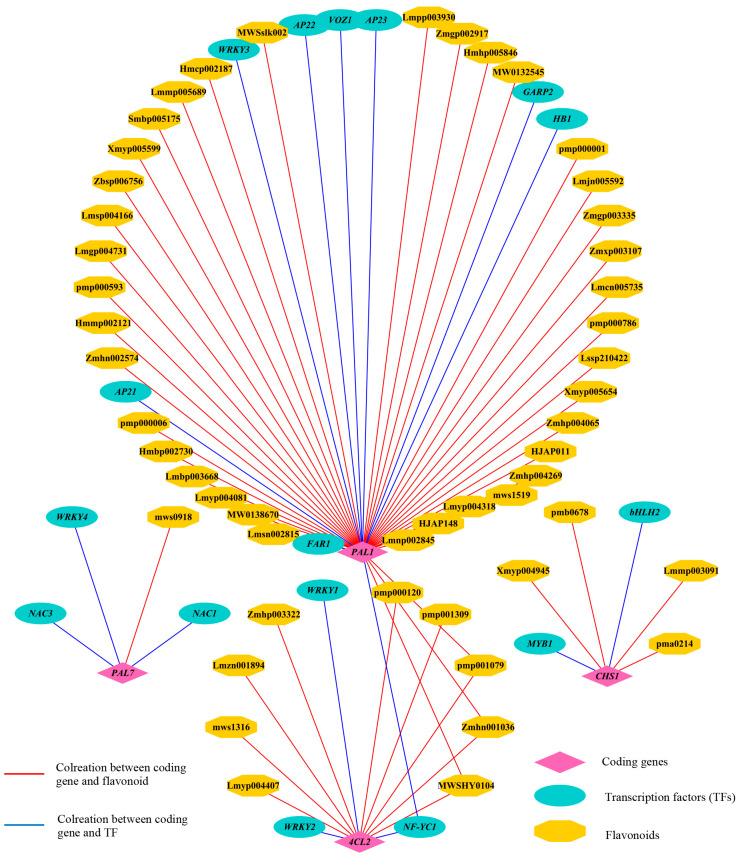
Transcriptional metabolic regulatory network of flavonoids in chaylte vines.

Figure 8 illustrates strong correlations between the four structural genes involved in the flavonoid biosynthesis pathway (*PAL1*, *PAL7*, *4CL2*, and *CHS1*) and 46 flavonoid metabolites. *PAL1*, an initiation gene for flavonoid biosynthesis, was found to be associated with 39 flavonoids and 9 transcription factors (TFs). It is noteworthy that the number of flavonoids and TFs associated with *PAL1* is significantly higher compared to the other PAL gene, *PAL7*. Similarly, *4CL2* and *CHS1* were both associated with 10 flavonoids and 6 transcription factors. This indicates that these four upstream structural genes, potentially regulated by specific TFs such as *NAC*, *MADS*, *WRKY*, and *MYB*, play a critical role in the biosynthesis of flavonoids in chaylte vines.

### 3.7. Determination of Total Flavonoid Content and Protective Enzyme Activity

**Figure 9:**
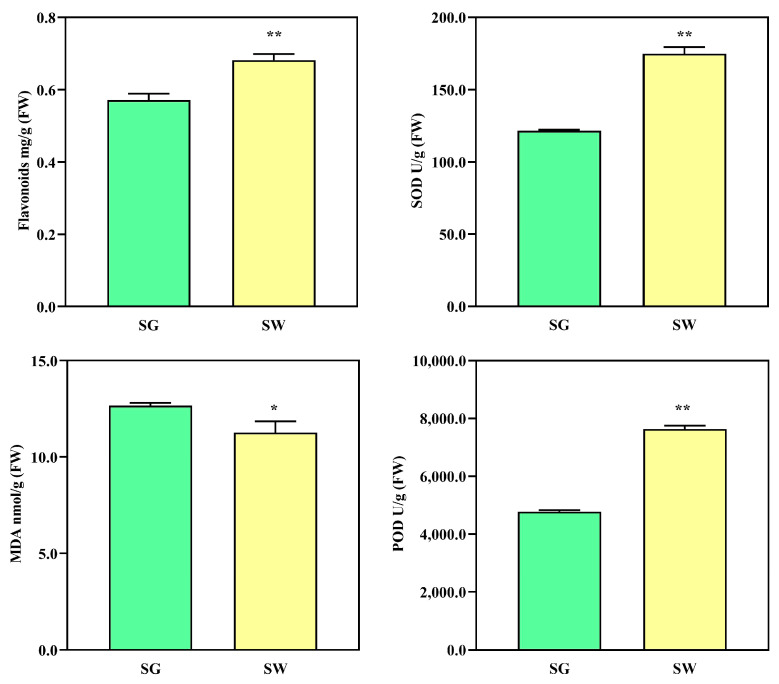
Determination of total flavonoid content and protective enzyme activity of two varieties of chaylte vines. * *p*-value was less than 0.05; ** *p*-value was less than 0.01.

The content of total flavonoids and the activity of the protective enzymes of two varieties of chaylte vines were determined to gain insights into physiological indicators (Figure 9). The results revealed that the content of total flavonoids in the SW samples (0.68 mg/g) was significantly higher compared to the SG samples (0.57 mg/g). Additionally, the SOD and POD activities of the SW samples were both significantly higher than those of the SG samples. Specifically, the SOD activity of the SW samples was 174.89 U/g, while that of the SG samples was only 121.51 U/g. Similarly, the POD activity of the SW samples was 7638.13 U/g, while that of the SG samples was only 4772.80 U/g. Additionally, the content of MDA in the SW samples (11.26 nmol/g) was significantly lower than that in the SG samples (12.67 nmol/g). These findings indicate that the SW samples exhibited a higher total flavonoid content and greater antioxidant capacity than the SG samples.

## 4. Discussion

### 4.1. Inconsistency in Total Flavonoid Content and Number of Flavonoids DAMs between Varieties

Compared to the SW samples, the SG samples had a lower total flavonoid content but higher amounts of their up-regulated flavonoid DAMs. This inconsistency was also reflected at the level of gene expression, where three PAL genes were significantly down-regulated, while one CHS gene and one 4CL gene were significantly up-regulated in the SG samples compared to the SW samples. It could be seen that the trends of PAL gene expression and total flavonoid content remained consistent between the two groups of samples, suggesting that the expression level of PAL-like genes might directly affect the accumulation of total flavonoids in the chaylte vines. It was not surprising to draw this inference because the biosynthesis of flavonoids in the plants was initiated by the biosynthesis of phenylpropanoid, and PAL, as the first key enzyme in the phenylpropanoid biosynthesis process, had an impact on all the downstream reactions in the flavonoid biosynthesis pathway [23]. An increase in PAL gene expression could cause the accumulation of flavonoids in plants [24,25,26].

In the present study, the expression trends of the 4CL and CHS genes were of interest, especially CHS. The CHS gene, located downstream of PAL, was responsible for catalyzing the production of chalcone from p-coumaroyl CoA, which formed the basic carbon skeleton of flavonoids [27]. As a result, CHS was considered to be the formal initiator of flavonoid biosynthesis [28]. In theory, changes in CHS gene expression should be influenced by the PAL gene and be consistent with the accumulation of total flavonoids. However, contrary to the norm, a member of the CHS gene family exhibited an expression trend opposite to what was typically observed in chaylte vines. From another perspective, the expression trend of this CHS gene was consistent with the change in the amount of flavonoid DAMs between the samples. This intriguing finding prompted a more detailed analysis of flavonoid DAMs between the two sample groups. The analysis revealed that not only was the amount of up-regulated flavonoid DAMs higher in the SG than in the SW but the distribution of flavonoid subclasses also differed between the two groups (Table 2). Among the 33 up-regulated flavonoid DAMs in the SW samples, 24 (72.73%) were flavones, while flavonols (24, 34.29%) dominated the 70 up-regulated flavonoid DAMs in the SG samples. Moreover, the number of other flavonoid subclasses, such as isoflavones, flavanones, and chalcones, was greater in the SG samples compared to the SW samples. Previous studies have demonstrated a positive correlation between the expression of CHS genes and the accumulation of flavonols and isoflavones in plants. Multiple studies have demonstrated that MYB-like transcription factors in soybeans can regulate the expression of CHSs genes, thereby influencing the accumulation of isoflavones [29,30,31]. Additionally, the transgenic plants of tomato showed an increased flavonol content due to the overexpression of the petunia CHS gene [32]. Therefore, the disparity in the number of flavonoid subclasses observed in different varieties of chaylte vines likely has some underlying connection with the differential expression of CHS genes.

In summary, the diversity of flavonoid subclasses in the SG samples was better than that of the SW samples despite the higher total flavonoid content in the SW samples. This inconsistency may be attributed to the differential expression of the PAL and CHS genes in the respective samples.

### 4.2. Inspiration for Breeding and Postharvest Preservation

Flavonoids possess significant antioxidant capacity, which plays a crucial role in preventing the postharvest aging of fruits and vegetables by increasing the antioxidant content and enzyme activity [23,33]. The key difficulty in the industrialization of the chaylte vine as a fresh vegetable lies precisely in its postharvest preservation and storage. Unlike the fruit (chayote), the freshness period of the chaylte vine is extremely short. After being picked, the chaylte vine quickly loses water, wilts on the shelves, and becomes commercially worthless. Therefore, breeding storage-resistant cultivars for the chaylte vine is an important breeding direction. Unfortunately, no relevant breeding and research work has been carried out on this matter. There are no reports available to reference regarding which varieties of chaylte vines are more resistant to storage after harvest.

Due to the absence of specialized cultivars on the market, coupled with the fact that the green-skinned variety is more widely cultivated, most of the existing chaylte vines have been produced by directly altering the cultivation of green-skinned fruiting *S. edule* [34]. However, the present study found that the white-skinned variety of the chaylte vine has a higher total flavonoid content and antioxidant capacity compared to the green-skinned variety. This suggests that the white-skinned variety may be more suitable for the production of storage-resistant chaylte vines than the widely cultivated green-skinned variety. Consequently, further research on the postharvest preservation of these two varieties could be conducted.

Moreover, attempts can be made to select and breed storage-resistant cultivars from white-skinned varieties in order to mitigate the postharvest problem. The substitution of varieties might have less impact on the consumers as there is no visible difference in the phenotype between the two varieties of chaylte vines. The findings of this study can offer valuable references for future research on the postharvest preservation and breeding of specialized cultivars of *S. edule* for chaylte vine production.

## 5. Conclusions

Although the total flavonoid content was lower, the SG samples had a greater diversity of flavonoid subclasses than the SW samples. This inconsistent finding was likely due to the differential expression of the PAL and CHS genes in the two varieties. Hence, it is suggested that the white-skinned variety may be more suitable for the production of storage-resistant chaylte vines than the widely cultivated green-skinned variety. These results, therefore, provide a basis for exploring the mechanisms behind flavonoid regulation and breeding specialized cultivars of *S. edule* for chaylte vine production.

## Figures and Tables

**Table 1:** RNA-seq data information of the chaylte vines.

Sample	Raw Reads	Clean Reads	Clean Base (G)	Q20 (%)	Q30 (%)	GC Content (%)	Unique Mapped
SG1	65,274,266	63,677,814	9.55	98.11	94.45	45.33	60,798,568 (95.48%)
SG2	64,068,410	62,111,032	9.32	98.04	94.35	45.42	59,252,336 (95.40%)
SG3	66,957,632	65,273,616	9.79	98.08	94.39	45.37	62,305,085 (95.45%)
SW1	55,198,928	53,479,702	8.02	98.1	94.45	45.09	50,993,963 (95.35%)
SW2	63,161,956	60,977,744	9.15	98.13	94.51	45.1	58,138,439 (95.34%)
SW3	55,068,678	52,936,534	7.94	98.11	94.51	45.16	50,476,417 (95.35%)

**Table 2:** Classification and number of flavonoid DAMs in SW samples compared to SG samples.

Subclasses	UP-Regulated	Down-Regulated	All
Flavones	24	23	47
Flavonols	5	24	29
Isoflavones	1	8	9
Flavanones	2	6	8
Chalcones	0	5	5
Flavanols	1	2	3
Flavanonols	0	1	1
Other Flavonoids	0	1	1
Total	33	70	103

## Data Availability

The sequencing data mentioned in this research can be downloaded from NCBI (www.ncbi.nlm.nih.gov). Number of bioproject: PRJNA1017555.

## Supplementary Materials

The following supporting information can be downloaded at https://www.mdpi.com/article/10.3390/cimb45110568/s1, Figure S1: TIC overlap plot of all samples (negative ion mode); Figure S2: TIC overlap plot of all samples (positive ion mode); Figure S3: Parameters and permutation test of OPLS-DA model; Table S1: Sequences of qRT-PCR primers of chaylte vines; Table S2: Methods for the determination of protective enzyme activity and MDA content; Table S3: All detected metabolites in chaylte vines of *S. edule*; Table S4: 277 DAMs in SW samples compared to SG samples; Table S5: 739 DEGs in SW samples compared to SG samples; Table S6: Genes involved in flavonoid-related pathways in Figure 5; Table S7: Transcription factors in quadrants 1, 3, 7, and 9.
