# Supplementary figures and images for: Multi-Omics Analyses Unravel Metabolic and Transcriptional Differences in Tender Shoots from Two Sechium edule Varieties
Source: Curr Issues Mol Biol. 2023 Nov 13;45(11):9060–75. doi: 10.3390/cimb45110568 (PMC10670898; doi:10.3390/cimb45110568)

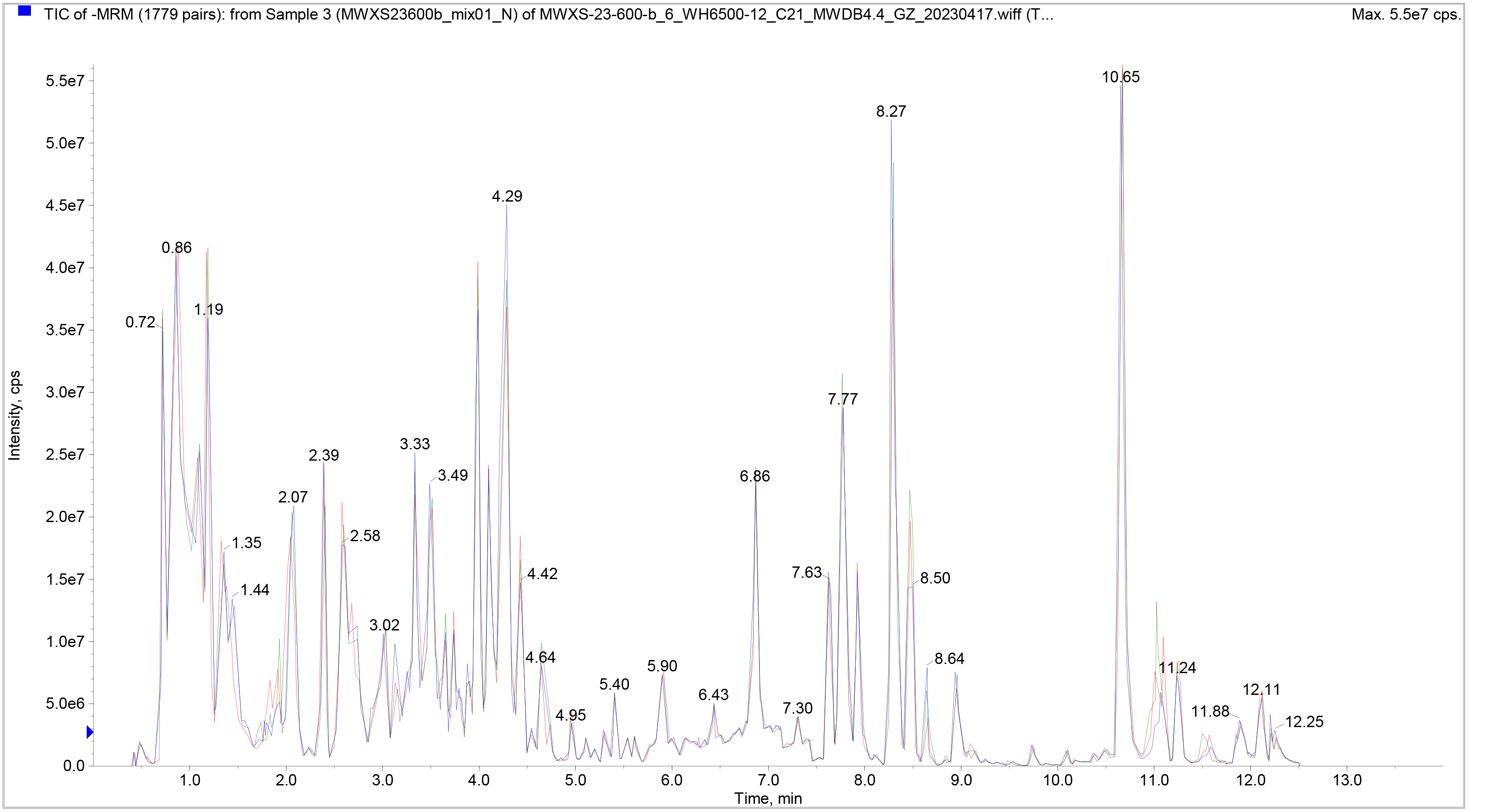

Supplement: Supplementary file 1 [file cimb-45-00568-s001.zip › FigureS1.tif]

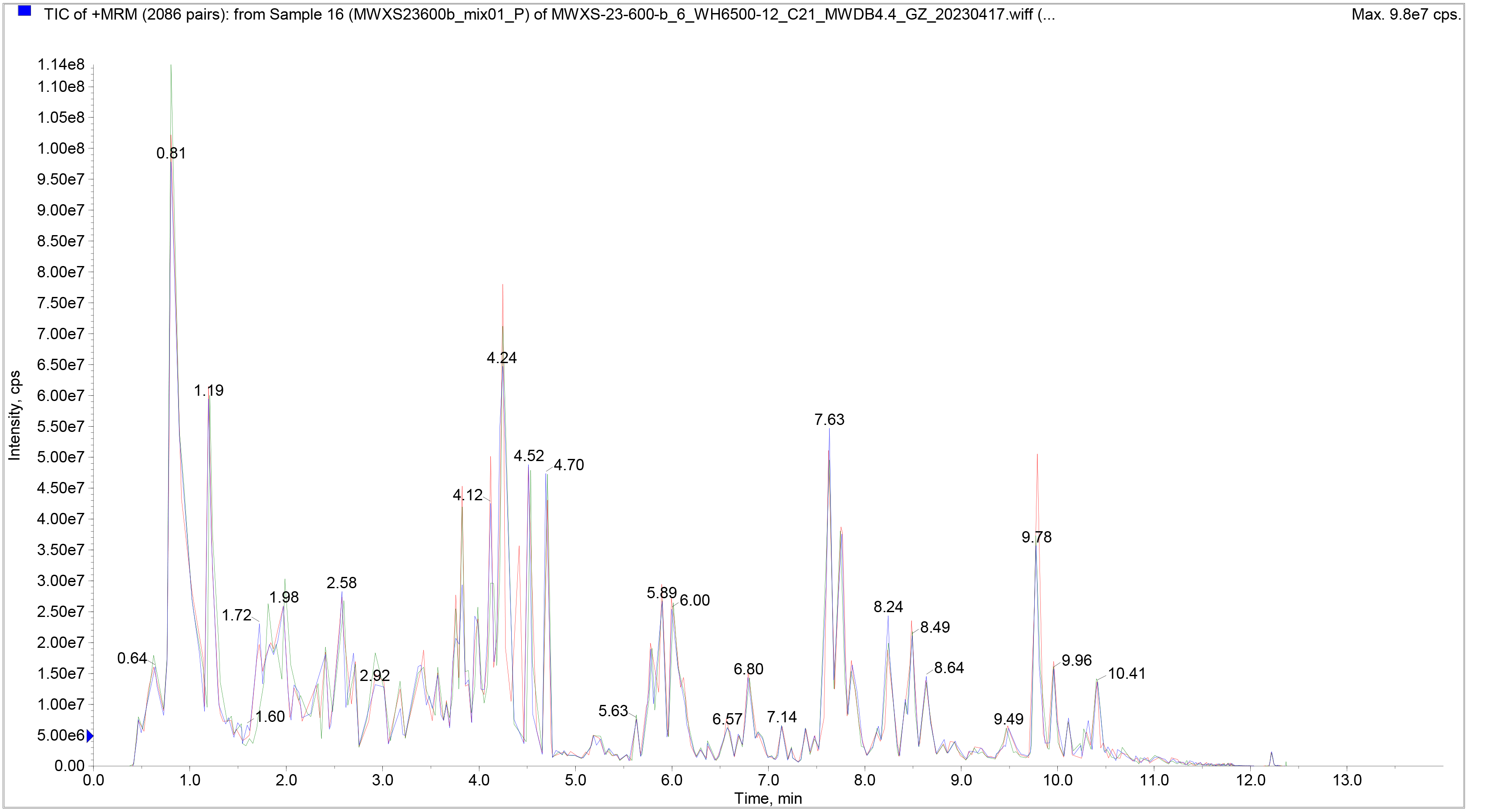

Supplement: Supplementary file 1 [file cimb-45-00568-s001.zip › FigureS2.tif]

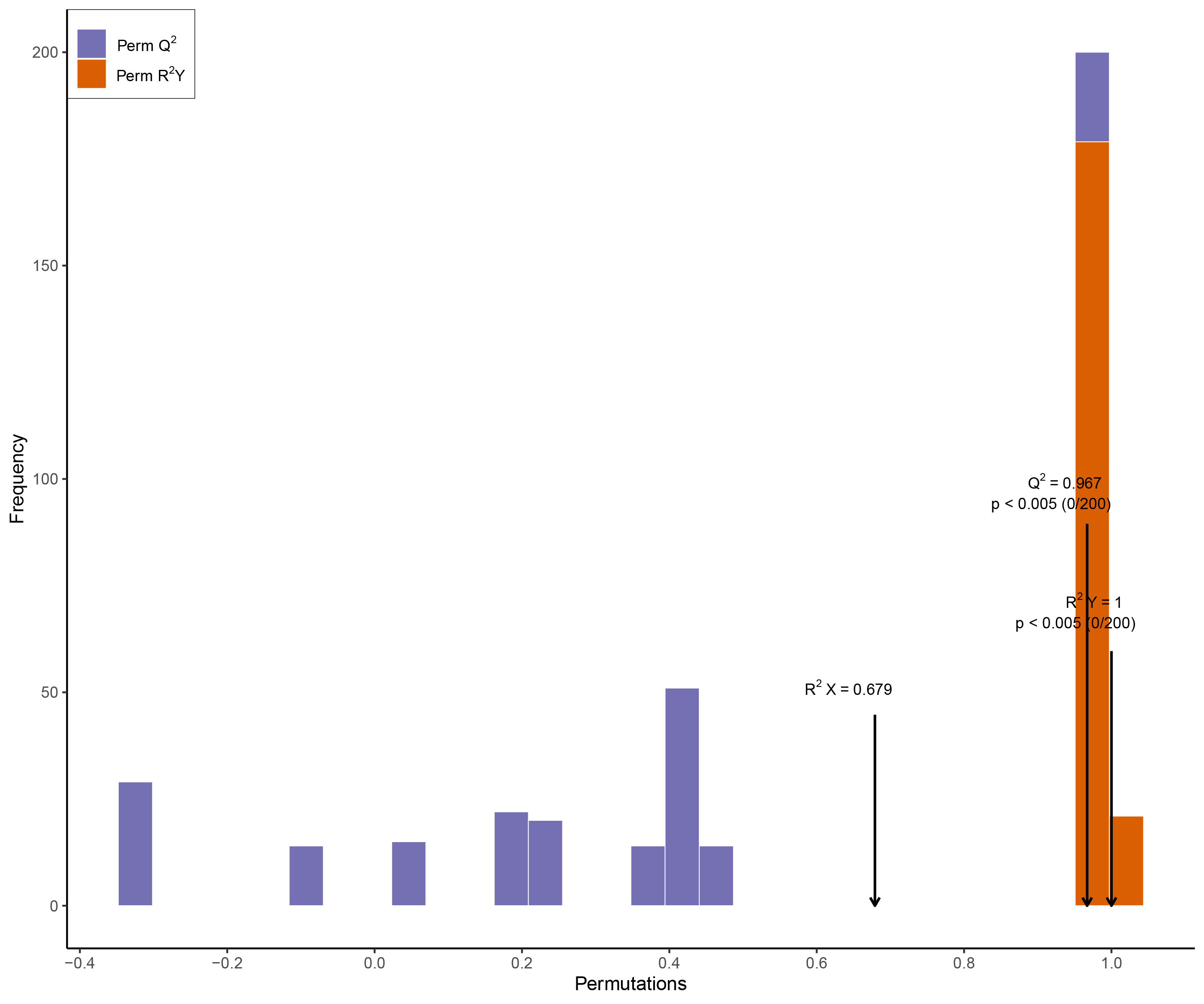

Supplement: Supplementary file 1 [file cimb-45-00568-s001.zip › FigureS3.tif]
